# A Challenging Case of Hepatoblastoma Concomitant with Autosomal Recessive Polycystic Kidney Disease and Caroli Syndrome—Review of the Literature

**DOI:** 10.3389/fped.2017.00114

**Published:** 2017-06-07

**Authors:** Nevil Kadakia, Steven J. Lobritto, Nadia Ovchinsky, Helen E. Remotti, Darrell J. Yamashiro, Jean C. Emond, Mercedes Martinez

**Affiliations:** ^1^Department of Pediatrics, Columbia University College of Physicians and Surgeons, New York, NY, United States; ^2^Department of Pediatrics, Children’s Hospital of Montefiore, Bronx, NY, United States; ^3^Department of Pathology and Cell Biology, Columbia University College of Physicians and Surgeons, New York, NY, United States; ^4^Department of Surgery, Columbia University College of Physicians and Surgeons, New York, NY, United States

**Keywords:** hepatorenal fibrocystic diseases, hepatoblastoma, autosomal recessive polycystic kidney disease, congenital hepatic fibrosis, Caroli syndrome

## Abstract

We report a rare case of an 18-month-old female with autosomal recessive polycystic kidney disease, Caroli syndrome, and pure fetal type hepatoblastoma. The liver tumor was surgically resected with no chemotherapy given. Now 9 years post resection she demonstrates no local or distant recurrence and stable renal function.

## Introduction

Hepatorenal fibrocystic diseases (HRFCDs) are a group of severe, monogenic disorders characterized by the common pathological appearance of developmental abnormalities mainly involving the liver and kidneys (1). Autosomal recessive polycystic kidney disease (ARPKD) is the most prevalent disorder affecting the kidneys, while congenital hepatic fibrosis (CHF) and Caroli syndrome are the most frequent anomalies found in the liver. ARPKD presents with progressive decline in renal function progressing to end stage kidney disease and large echogenic kidneys on the sonogram (1). CHF is characterized by biliary ductal plate malformation, abnormal portal vein, and progressive fibrosis. Caroli disease is characterized by a saccular or fusiform dilation of medium or large bile ducts, and when present in combination with CHF is called Caroli syndrome. Hepatoblastoma (HB) is the most prevalent primary hepatic tumor in children, usually presenting before 5 years of age. Although the etiology is unknown, it is associated with prematurity, low birth weight, and certain genetic disorders including familial adenomatous polyposis, Beckwith–Wiedemann syndrome, hemihypertrophy (2), and rarely with glomerulocystic kidney disease (GCKD) (3) and ARPKD (4). A combination of chemotherapy and surgical resection is required for the treatment of HB, except for the pure fetal type that can be cured with primary resection (5–7). Renal injury could be exacerbated by the toxicity of chemotherapy agent especially in patients with preexisting renal dysfunction. We report an unusual case of a young girl who presented with coexisting ARPKD, Caroli syndrome, and HB, whose malignancy was cured with surgical resection without chemotherapy, thereby emphasizing the importance of limiting renal injury in a patient with ARPKD.

## Background

A 2-month-old infant presented with hepatosplenomegaly. Sonographic imaging showed large kidneys with multiple cysts, multiple liver cysts, splenomegaly, and no ascites. Her laboratory results included a creatinine clearance of 43.33 ml/min/1.73 m^2^, normal liver enzymes, and coagulation factors. Serum alpha fetoprotein (AFP) level, checked because of her firm palpable liver, was 500 ng/ml (considered normal for age). Her platelet count was 250,000. She was diagnosed with ARPKD and CHF, based on clinical presentation and imaging. Blood pressure was checked as part of the evaluation for renal dysfunction and found to be elevated, despite no symptoms. Endoscopy was not performed. Genetic testing demonstrated two mutations associated with PKHD-1: a novel missense mutation c.1616T>C (p.Ile539Thr) in exon 18 and a frameshift mutation c.3761delCCinsG (p.Ala1254fs) in exon 32.

At 18-month follow-up, her AFP level rose to 800 ng/ml and an abdominal sonogram demonstrated a new 4.5 cm hyperechoic mass in the right lobe of the liver with involvement of the caudate. She was offered liver biopsy and primary chemotherapy (in her native country). Primary resection was not pursued due to concerns that after resection residual hepatocyte mass would be insufficient to maintain adequate liver function.

**Figure 1:**
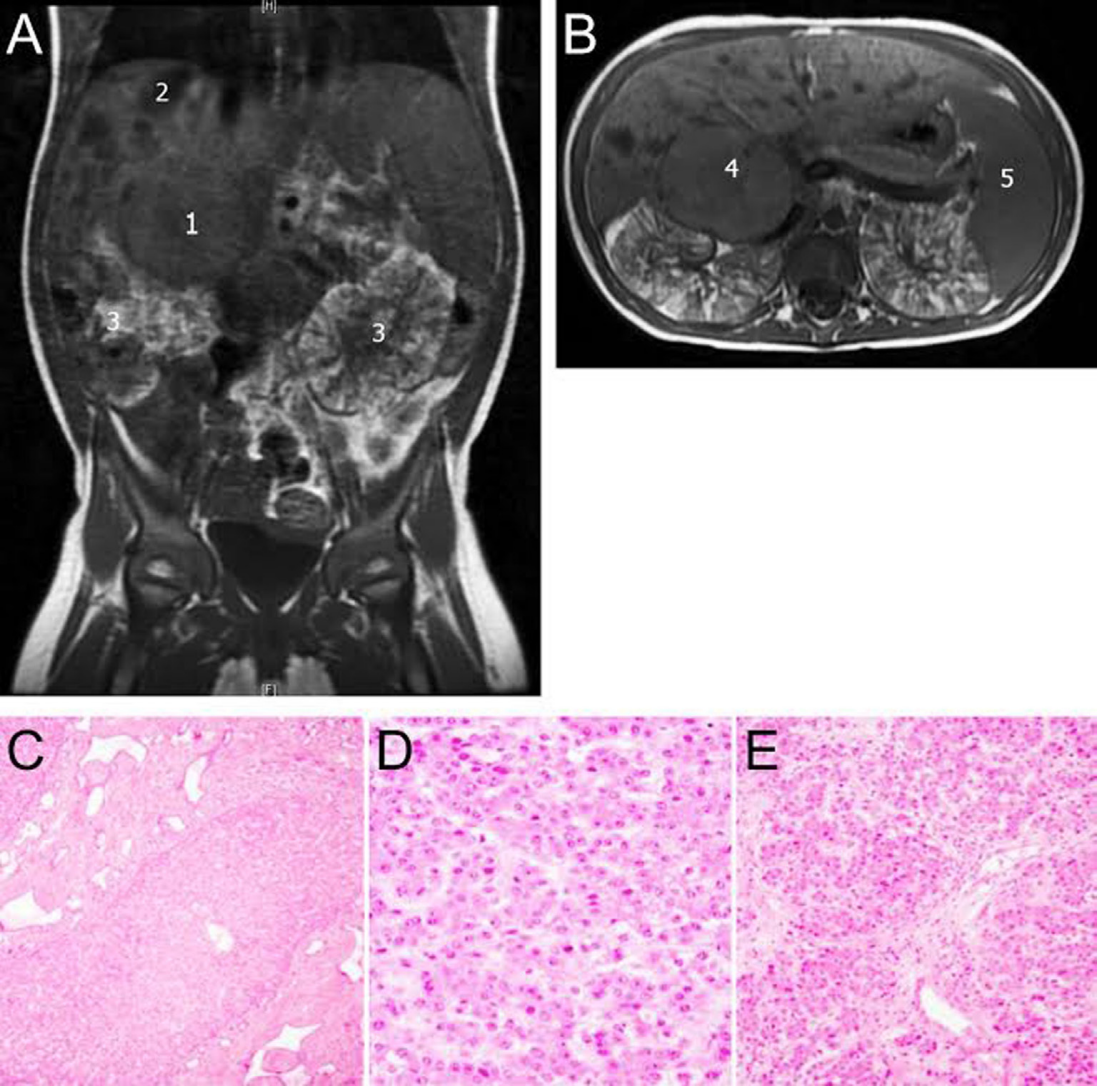
**(A,B)** Non-contrast MRI abdomen 1,4: hepatoblastoma (HB), 2: liver cysts, 3: enlarged polycystic and distorted kidneys, and 5: enlarged spleen. Lower panel: histological features of tumor and congenital hepatic fibrosis (CHF). **(C)** Liver demonstrating aberrant ductular proliferation and portal fibrosis characteristic of a bile ductal plate malformation, supporting the diagnosis of CHF. **(D,E)** Liver tissue representing pure fetal-type HB with low mitotic activity.

She presented to our center at 22 months of age for a second opinion. Her AFP was 1,200 ng/ml and a non-contrast MRI (secondary to impaired renal function; Figure 1) revealed a 4.6 cm × 3.9 cm heterogeneous mass in the right hepatic lobe near segment 5 with possible involvement of the caudate. Assuming the tumor to be HB with two adjacent sectors of the liver tumor-free and no lymph node or vascular involvement, it would be staged as Pretext 2 (8). Imaging also demonstrated dilatation of the bile ducts and focal ectasia consistent with Caroli syndrome. Liver synthetic function was normal, and no clinical signs of portal hypertension were appreciated before the resection. The consensus was to perform a right hepatectomy even after acknowledging the possibility of postresection liver dysfunction since the remaining liver tissue was not normal. Given this risk, the child’s father was evaluated as a potential living liver donor should rescue transplant be necessary. Fortunately, she tolerated the procedure without evidence of posthepatectomy liver dysfunction or other postoperative complications. Her creatinine clearance was 52 ml/min/1.73 m^2^ before and 47.5 ml/min/1.73 m^2^ after the surgery. Histopathology of the resected specimen revealed pure fetal epithelial-type HB with low mitotic activity (Figure 1). The neoplasm was composed of tumor cells resembling fetal hepatocytes with mild nuclear enlargement compared to non-neoplastic hepatocytes. The low mitotic activity correlated with a Ki67 proliferative index of 1%. A reticulin stain showed decreased staining in the tumor and highlighted the focal microtrabecular architecture with increased thickness of liver cell plates. Focal extramedullary hematopoiesis was present. There was no microscopic vascular invasion, and the resection margins were free of tumor. The non-neoplastic liver was remarkable for changes consistent with CHF. Given the favorable histopathology of the pure fetal epithelial-type HB, no adjuvant chemotherapy was administered.

At 6 years of age, the patient presented with signs of portal hypertension related to her CHF manifested as esophageal varices managed medically and endoscopically. As expected, liver synthetic function was preserved and hypersplenism was evident. During 9 years of follow-up post resection, her serum AFP remained <3 mg/dl and serial subsequent MRI images indicated no recurrent tumor. At the age of 11 years, her creatinine clearance was 31.2 ml/min/1.73 m^2^, with good overall growth and development. Her most recent platelet count was 35,000, liver function is normal, and no hepatic inflammation is evident.

## Discussion

We report a case of a child presenting with an unusual association of ARPKD, Caroli Syndrome, and HB. HB is primarily a disease of WNT pathway activation and CTNNB1 mutations that occur in 70% of cases. The annual incidence in the United States has doubled from 0.8 (1975–1983) to 1.6 (2002–2009) per 1 million in children younger than 19 years (9). The response to treatment, recurrence, and overall prognosis of HB depends on the histological classification and resectability of the tumor. Small series reports have suggested that pure fetal-type HB has a significantly higher cure rate than anaplastic or poorly differentiated small cell histology (6). Although surgical resection is the cornerstone of cure, neoadjuvant chemotherapy is administered to improve the resectability of large tumors, and adjuvant chemotherapy is administered after resection to prevent recurrence in most cases except for the pure fetal variant.

Autosomal recessive polycystic kidney disease and GCKD are part of a clinical spectrum known as HRFCDs; these conditions are linked to genetic mutations resulting in developmental abnormalities of the liver, kidneys, lungs, bones, and central nervous system (10). ARPKD has estimated incidence of 1/20,000 and is caused by mutations in the PKHD1 gene, located on chromosome 6p21 and extending over 470 kb of the genomic sequence (11), CHF is an autosomal recessive-inherited malformation. It is defined pathologically by variable degrees of periportal fibrosis and irregularly shaped proliferating bile ducts. These changes manifest clinically as signs or symptoms related to progressive portal hypertension in affected patients (12).

The incidence of concurrent ARPKD, CHF, and HB is rare making universal screening seem cost ineffective. Unfortunately, there are no clinical clues to the development of HB in this setting other than serial AFP assessments and imaging. However, early detection as in our case could permit an aggressive surgical approach sparing precious remaining kidney reserve from chemotoxicity and liver transplantation. So, the benefit of screening to the individual may justify the costs involved.

Although the progression of renal and liver diseases varies, the majority of the patients with ARPKD will develop renal dysfunction requiring renal replacement therapy and ultimately transplantation. Cisplatin is one of the most active chemotherapy agents for HB (13) but is potentially nephrotoxic. Administration of this agent is challenging in this population with preexisting renal compromise. This fact is illustrated in the literature review of six previous reported cases of HRFCD and HB (3, 4, 14–17) (Table 1). In these reports, four of six patients developed renal failure with significant contribution to overall morbidity and mortality. Notably, only one patient survived initial chemotherapy and resection, requiring dialysis 10 months after completion of treatment (4). One case report of surgery alone to treat a large tumor resulted in recurrence and death by 11 months, but no justification was stated for the approach used (14). Our patient is the only reported case with this association that survived curative treatment of HB with preserved renal function and no renal replacement therapy or transplantation. We attribute this success to the therapeutic approach of aggressive early resection and avoidance of chemotherapy.

**Table 1:** **Summary of previous case reports**.

Case report	Renal defects	Location of hepatoblastoma	Age at Dx in months	Treatment modality	Outcome
Reference					
Rao et al. (14)	Multicystic	R lobe (15 cm × 8 cm)	24	R hepatectomy	Died of recurrent disease 11 months after resection
	R kidney	Fetal variant			
Bhaskar et al. (15)	GCKD	L lobe (6 cm × 5 cm × 4 cm)	3	No specific therapy	RF. Died of sepsis soon after presentation at 3-month olds
Greer et al. (16)	GCKD	R lobe (5 cm × 5 cm)	2	No reported	RF. No reported survival outcome
Kummerfeld et al. (4)	ARPKD	L lobe (6 cm)	18	Chemotherapy, L hepatectomy	Survived. RF required RRT (10 months)
Luoto et al. (17)	ARPKD	Multifocal	62	Liver transplant	Died of recurrence
Zaman et al. (3)	GCKD	R lobe (7 cm × 5 cm)	5	Chemotherapy	RF and died
2016 (this case)	ARPKD	R lobe (5 cm × 4 cm)	24	R hepatectomy. No chemotherapy	Survived. Stable renal function

*ARPKD, autosomal recessive polycystic kidney disease; GCKD, glomerulocystic kidney disease; Dx, diagnosis; L, left; R, right; RF, renal failure; RRT, renal replacement therapy*.

Nevertheless, this approach should be reserved for selected patients who qualify for primary surgery based on PRETEXT classification. We believe that these patients should be referred to centers with surgical expertise in tumor management and solid organ transplantation. Complications of hepatic surgery in children with HB are associated with significant morbidity and mortality rates, reducing the 5-year overall survival from 72 to 50% in high-risk groups (18), despite advances in operative techniques and perioperative care.

The surgical approaches to HB differ between European and North American centers. The Société Internationale d’Oncologie Pédiatrique—Epithelial Liver Tumor Study group, representing European centers, have used a single strategy for all patients including neoadjuvant chemotherapy followed by surgical intervention regardless of size or histology, in order to make the tumor smaller, more delimited from the surrounding hepatic parenchyma and more likely to be completely resected. In contrast, the children’s oncology group, representing North American centers have offered resection at diagnosis whenever prudently feasible followed by adjuvant chemotherapy as a strategy to minimize overall chemotherapy exposure and toxicity (19). The decision for upfront resection is determined by location and resectability of the tumor with a clear margin and not by tumor size (5). Overall outcome has been similar, despite these different approaches. Our case had favorable histology (fetal type), location amenable to primary resection, and significant risk of morbidity from the administration of chemotherapy due to preexisting renal disease leading to the approach recommended.

## Conclusion

This case represents a successful approach for the treatment of an unusual combination of HRFCD and HB. The rarity of this association warrants this report and merits meticulous genetic investigation to discover factors predisposing to both entities. The case also illustrates a personalized treatment approach based on a particular clinical situation taking into account all organ systems involved and resulted in an excellent outcome compared to similar cases in the literature treated with traditional approaches. Standardized approaches to specific tumors remain prudent; however, alternative therapies in patients with malignancies and impaired renal function taking into account the nephrotoxicity of the treatment regimen merit equal consideration. Although no formal recommendation can be drawn from a few case reports, we feel that given the reported association, screening for HB using AFP and imaging of the liver could be considered in patients with ARPKD and CHF, to warrant early detection of tumor. This practice could permit an individualized approach using upfront resection and elimination/minimization of chemotherapy.

## Ethics Statement

This is a case report and does not require committee approval. The authors communicate with the parents of the minor and got verbal consent to publish this report. This report does not include any identifiers of the patient to protect patient confidentiality.

## Author Contributions

All the authors made a great effort to build this novel case report. NK being the first author, constructed a draft based on the patient’s case presentation and history. SL, NO, HR, DY, and JE added the literature review in the draft and made necessary changes in the draft accordingly, making it more concise. MM, being the corresponding author design the case report, supervise each step during the writing of this case report, took a remarkable effort to enhance the quality of the manuscript since the beginning, and made the final assessment as well.

## Conflict of Interest Statement

The authors declare that the research was conducted in the absence of any commercial or financial relationships that could be construed as a potential conflict of interest.

## References

[1] Koh KN Park M Kim BE Bae KW Kim KM Im HJ Prognostic implications of serum alpha-fetoprotein response during treatment of hepatoblastoma. Pediatr Blood Cancer (2011) 57(4):554–60. 10.1002/pbc.23069 21370433

[2] Tomlinson GE Kappler R. Genetics and epigenetics of hepatoblastoma. Pediatr Blood Cancer (2012) 59(5):785–92. 10.1002/pbc.24213 22807084

[3] Zaman R Maggi A Rajpoot SK Joshi DD. Glomerulocystic kidney disease and hepatoblastoma in an infant: a rare presentation. Case Rep Nephrol Dial (2015) 5(3):200–3. 10.1159/000439520 26688803 PMC4677724

[4] Kummerfeld M Klaunick G Druckler E Classen CF Hauenstein C Stuhldreier G. Hepatoblastoma in association with bilateral polycystic kidneys. J Pediatr Surg (2010) 45(11):e23–5. 10.1016/j.jpedsurg.2010.07.018 21034924

[5] Maibach R Roebuck D Brugieres L Capra M Brock P Dall’Igna P Prognostic stratification for children with hepatoblastoma: the SIOPEL experience. Eur J Cancer (2012) 48(10):1543–9. 10.1016/j.ejca.2011.12.011 22244829

[6] Qiao GL Chen Z Wang C Ge J Zhang Z Li L Pure fetal histology subtype was associated with better prognosis of children with hepatoblastoma: a Chinese population-based study. J Gastroenterol Hepatol (2016) 31(3):621–7. 10.1111/jgh.13165 26401976

[7] Malogolowkin MH Katzenstein HM Meyers RL Krailo MD Rowland JM Haas J Complete surgical resection is curative for children with hepatoblastoma with pure fetal histology: a report from the Children’s Oncology Group. J Clin Oncol (2011) 29(24):3301–6. 10.1200/JCO.2010.29.3837 21768450 PMC3158601

[8] Czauderna P Haeberle B Hiyama E Rangaswami A Krailo M Maibach R The Children’s Hepatic Tumors International Collaboration (CHIC): novel global rare tumor database yields new prognostic factors in hepatoblastoma and becomes a research model. Eur J Cancer (2016) 52:92–101. 10.1016/j.ejca.2015.09.023 26655560 PMC5141607

[9] PDQ Pediatric Treatment Editorial Board. Childhood liver cancer treatment (PDQ^®^): health professional version. PDQ Cancer Information Summaries [Internet]. Bethesda, MD: National Cancer Institute (US) (2002). Available from: http://www.ncbi.nlm.nih.gov/books/NBK65790/

[10] Johnson CA Gissen P Sergi C Molecular pathology and genetics of congenital hepatorenal fibrocystic syndromes. J Med Genet (2003) 40(5):311–9. 10.1136/jmg.40.5.311 12746391 PMC1735460

[11] Obeidova L Seeman T Elisakova V Reiterova J Puchmajerova A Stekrova J. Molecular genetic analysis of PKHD1 by next-generation sequencing in Czech families with autosomal recessive polycystic kidney disease. BMC Med Genet (2015) 16:116. 10.1186/s12881-015-0261-3 26695994 PMC4689053

[12] Bayraktar Y Yonem O Varli K Taylan H Shorbagi A Sokmensuer C. Novel variant syndrome associated with congenital hepatic fibrosis. World J Clin Cases (2015) 3(10):904–10. 10.12998/wjcc.v3.i10.904 26488028 PMC4607810

[13] Black CT Cangir A Choroszy M Andrassy RJ. Marked response to preoperative high-dose cis-platinum in children with unresectable hepatoblastoma. J Pediatr Surg (1991) 26(9):1070–3. 10.1016/0022-3468(91)90676-K 1658288

[14] Rao PS Krishna A Rohatgi M Multicystic kidney in association with hepatoblastoma – a case report. Jpn J Surg (1989) 19(5):583–5. 10.1007/BF02471667 2556604

[15] Bhaskar KV Joshi K Banerjee CK Hepatoblastoma with glomerulocystic disease – a mere coincidence or an association? Nephron (1990) 54(3):273–4. 10.1159/000185872 2156184

[16] Greer ML Danin J Lamont AC. Glomerulocystic disease with hepatoblastoma in a neonate: a case report. Pediatr Radiol (1998) 28(9):703–5. 10.1007/s002470050444 9732498

[17] Luoto TT Pakarinen MP Jahnukainen T Jalanko H. Liver disease in autosomal recessive polycystic kidney disease: clinical characteristics and management in relation to renal failure. J Pediatr Gastroenterol Nutr (2014) 59(2):190–6. 10.1097/MPG.0000000000000422 24806835

[18] Becker K Furch C Schmid I von Schweinitz D Haberle B. Impact of postoperative complications on overall survival of patients with hepatoblastoma. Pediatr Blood Cancer (2015) 62(1):24–8. 10.1002/pbc.25240 25251521

[19] Aronson DC Meyers RL. Malignant tumors of the liver in children. Semin Pediatr Surg (2016) 25(5):265–75. 10.1053/j.sempedsurg.2016.09.002 27955729

